# Estradiol and the Development of the Cerebral Cortex: An Unexpected Role?

**DOI:** 10.3389/fnins.2018.00245

**Published:** 2018-05-25

**Authors:** Matthew C. S. Denley, Nicholas J. F. Gatford, Katherine J. Sellers, Deepak P. Srivastava

**Affiliations:** ^1^Department of Basic and Clinical Neuroscience, Maurice Wohl Clinical Neuroscience Institute, London, United Kingdom; ^2^MRC Centre for Neurodevelopmental Disorders, King's College London, London, United Kingdom

**Keywords:** aromatase, subventricular zone, cortical plate, neurogenesis, migration, sexual dimorphism, 17β-estradiol, brain synthesized

## Abstract

The cerebral cortex undergoes rapid folding in an “inside-outside” manner during embryonic development resulting in the establishment of six discrete cortical layers. This unique cytoarchitecture occurs via the coordinated processes of neurogenesis and cell migration. In addition, these processes are fine-tuned by a number of extracellular cues, which exert their effects by regulating intracellular signaling pathways. Interestingly, multiple brain regions have been shown to develop in a sexually dimorphic manner. In many cases, estrogens have been demonstrated to play an integral role in mediating these sexual dimorphisms in both males and females. Indeed, 17β-estradiol, the main biologically active estrogen, plays a critical organizational role during early brain development and has been shown to be pivotal in the sexually dimorphic development and regulation of the neural circuitry underlying sex-typical and socio-aggressive behaviors in males and females. However, whether and how estrogens, and 17β-estradiol in particular, regulate the development of the cerebral cortex is less well understood. In this review, we outline the evidence that estrogens are not only present but are engaged and regulate molecular machinery required for the fine-tuning of processes central to the cortex. We discuss how estrogens are thought to regulate the function of key molecular players and signaling pathways involved in corticogenesis, and where possible, highlight if these processes are sexually dimorphic. Collectively, we hope this review highlights the need to consider how estrogens may influence the development of brain regions directly involved in the sex-typical and socio-aggressive behaviors as well as development of sexually dimorphic regions such as the cerebral cortex.

## Introduction

**Figure 1:**
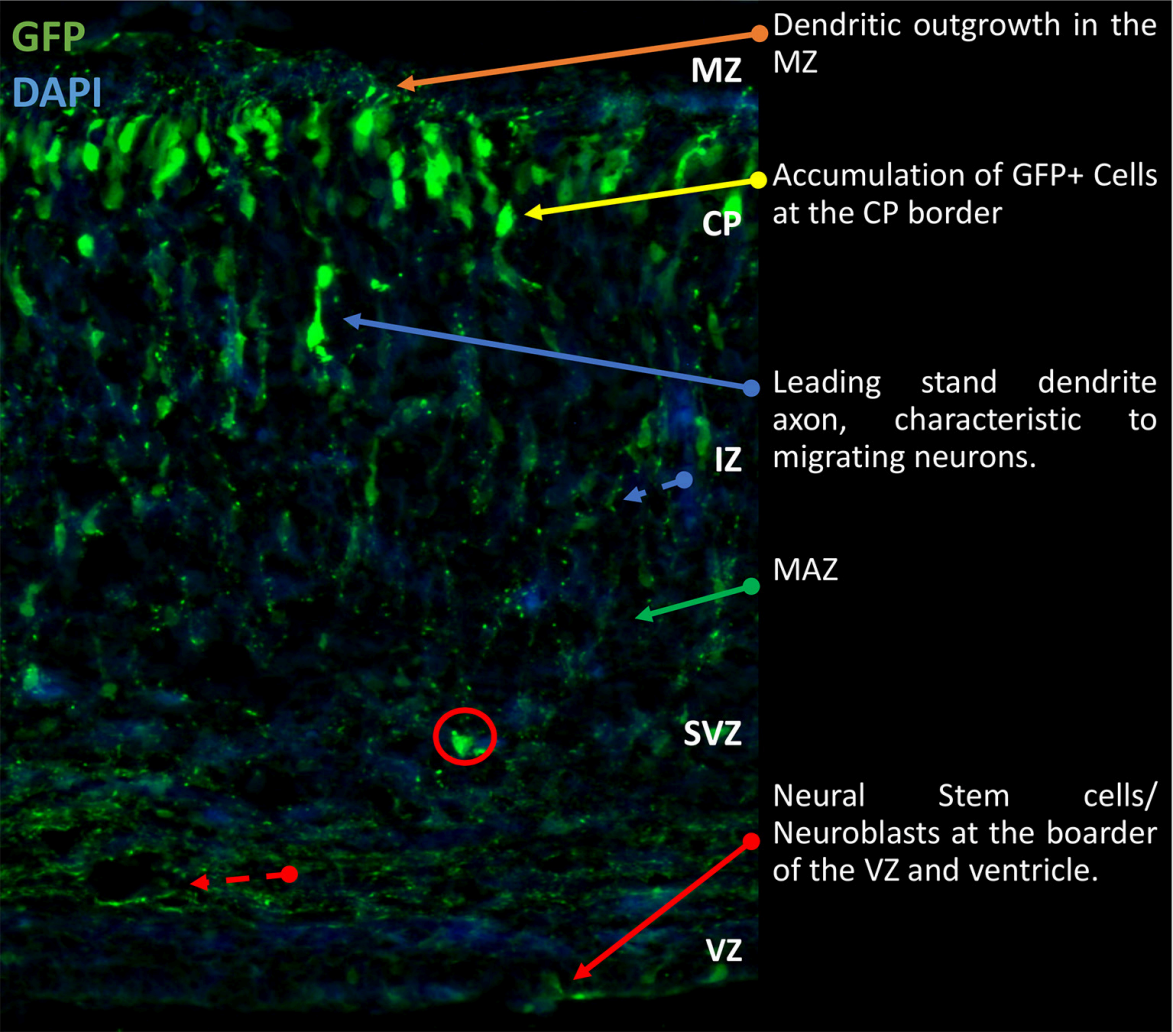
Gross morphological schematic of sub-compartments in the developing rodent cortex. Representative image of developing cortex. Electroporation of eGFP was performed at E14.5 and brains collected at P0 as previously described (Srivastava et al., 2012a,b). The cortex is comprised of four morphologically distinct regions, the VZ, SVZ, IZ, and CP. Further to this there are the MAZ and MZ, located in the IZ and CP respectively. Located on the basal surface of the cortex proximal to the cerebral ventricles is the VZ responsible for generation of NSCs. Beyond the VZ, the SVZ contains proliferating and early differentiating neural progenitors. Between the SVZ and IZ, the MAZ is a point of accumulation of polarizing cells. After which the cells migrate through the IZ to the CP where terminal translocation takes place. This brief outline is the general schematic throughout development of the cortex. Cells migrate to the outmost layer and continually build on top of each other in a sedimentary manner. IZ, intermediate zone; MAZ, multipolar cell accumulation zone; CP, cortical plate; GFP, green fluorescence; NSCs, Neural Stem Cells; VZ, ventricular zone; SVZ, subventricular zone; MZ, marginal zone.

The complex neuronal organization and architecture of the cerebral cortex is thought to be responsible for the higher cognitive function bestowed upon mammals. The unique anatomical compartmentalization and lamination of discrete neurons arranges into horizontal layer identifiable with specific molecular markers (Kriegstein and Parnavelas, 2006; Molyneaux et al., 2007; Greig et al., 2013). This organization is established during embryonic development, and is achieved in an “inside–outside” fashion, with the “deep” or “inner” layers developing first and the outer layers developing last. Critically, this unique cytoarchitecture is the basis by which the correct assembly of synaptic connectivity and therefore, functional cortical circuitry is established (Marín et al., 2010). Mechanistically, the inside-outside organization of the cerebral cortex is established throughout development and controlled by coordinating processes in neurogenesis, cell migration (Götz and Huttner, 2005; Taverna et al., 2014) as well as responses to extracellular cues and activation of intracellular signaling pathways (Hippenmeyer, 2014; Hansen et al., 2017). The phenotypic display of this coordination can be seen in Figure 1. Whilst our understanding of the molecular events that underlie these processes is ever growing, understanding the mechanisms and signals that exert influences over corticogenesis remains a major challenge. This is particularly emphasized by increasing evidence that abnormal corticogenesis may contribute to a range of neurodevelopment and psychiatric disorders (Hoerder-Suabedissen et al., 2013; Ishii et al., 2016).

## Background

Neurons forming the basic unit of nervous tissue architecture was a notion first proposed and described by Ramón y Cajal (1911). Connectivity between these units underlies the normal brain functions of neural circuitry in cognition and behavior. Therefore, understanding the developmental formation of neural circuitry is vital to understanding how these structures respond to environmental, physiological and pathological stimuli. Steroid hormones, such as estrogen, have long been the focus of research and review (Alcaraz et al., 1969; Dominique Toran-Allerand, 1976; Hammond and Rowe, 1976), and have long been suggested to be key signals orchestrating the development of sexual dimorphism of many cerebral regions. However, previous research has also indicated that estrogen can have wildly polarized effects in terms of behavior and physiology (Alcaraz et al., 1969; Gillies and McArthur, 2010). Research has also suggested that this response is due to specific estrogen receptor subtypes and, thus, is dependent on the expression in the discrete location of the receptor in specific brain regions. However, response to estrogen exposure is impeccably sensitive to factors such as dose and stage of life cycle (Katzenellenbogen et al., 2000; Sánchez-Criado et al., 2004). More recent research has made attempts to explain the mechanism behind sexual dimorphic response to estrogen during development (Grassi D. et al., 2012; Grassi S. et al., 2012; Liu et al., 2017). Interest in estrogen is further emphasized because of the demonstrable ability to affect psychiatric and neurodegenerative disorders (Arevalo et al., 2011; Srivastava et al., 2013b; Gobinath et al., 2017). This includes, for example, Autism Spectrum Disorder, which shows a strong male bias (Werling and Geschwind, 2013) and therefore suggests sexual dimorphic underlying physiology (Adhya et al., 2018).

Sexual dimorphism is not wholly dependent on the action and mechanism of hormones. Indeed, sex hormones and other gonadal secretions do have sex-specific effects on the brain, but gene expression in individual cells may have a larger role in the dimorphic phenotype. Increasing evidence has suggested a role for developmental mediation by X and Y chromosome-linked genes (Arnold, 2004). These revelations have led to a neurodevelopmental debate: epigenetics or genetics? This debate suggested that development of specific areas in the brain may be hormone-dependent, whilst others are hormone-independent (Reisert and Pilgrim, 1991). Here, we specifically discuss the hormone-dependent roles in neurodevelopment.

Estrogens are a class of steroids of which 17β-estradiol (often referred to as estradiol or just E2) is the main biologically active form (Blaustein, 2008). This class of steroid has long been known to exert powerful effects on development of the nervous system, as well as its function and plasticity (McEwen and Alves, 1999; McCarthy, 2008; Brinton, 2009; Srivastava et al., 2013b). Importantly, increasing evidence suggests that in addition to their actions within the hypothalamus (Kelly et al., 2005), estrogens exert effects within the neocortex and hippocampus (McEwen and Alves, 1999; Srivastava et al., 2013b). Moreover, the actions of estrogens during development and in the mature brain are not limited to those described in females. Effects of estrogens have also been consistently reported in males, albeit in a sexually dimorphic manner in many cases (Gillies and McArthur, 2010; McCarthy and Nugent, 2013; Gobinath et al., 2017). However, our current understanding of the role estrogens such as estradiol play in regulating the cellular or molecular processes critical for corticogenesis is limited. In this review, we will discuss the evidence that implicate a role for estrogens during the development of the cortex. Furthermore, we will highlight specific molecular pathways involved in developing the complex neuronal organization of the neocortex and emphasize how estrogens, particularly, estradiol signaling, interacts with these processes.

## The case for estrogen in the developing cortex

Canon has long established that the main source of estrogen production is the female sex organs. However, there is growing evidence that the key enzyme that converts androgens into estrogens, aromatase, is highly expressed in nervous tissue (Yague et al., 2006; Ish et al., 2007; Hojo et al., 2009; Boon et al., 2010; Saldanha et al., 2013). Aromatase has been identified in the hypothalamus, hippocampus, visual cortex, and temporal cortex in avian, mammalian, and human brain (Rune and Frotscher, 2005; Yague et al., 2006; Boon et al., 2010; Azcoitia et al., 2011; Saldanha et al., 2011).

The abundance of information on aromatase expression hints toward its fundamental importance. Aromatase expression has been evolutionarily conserved in nervous tissue, from chordates and early teleost. Complete removal of sex organs demonstrates that the expression is indeed sourced from within the nervous tissue, as removal does not lead to a complete loss-of-function (Yague et al., 2006; Ish et al., 2007; Hojo et al., 2009; Boon et al., 2010; Konkle and McCarthy, 2011; Saldanha et al., 2011). Indeed, Callard et al. (1978) highlighted that aromatase expression is highly concentrated to the forebrain in vertebrates. Furthermore, goldfish and toadfish demonstrate a high concentration of aromatase in the hypothalamus and preoptic areas (Pasmanik and Callard, 1985). Interestingly, work in toadfish demonstrated that expression of aromatase in certain neural areas accounted for sexually dimorphic behaviors (Pasmanik and Callard, 1985). Estrogens and aromatase expression in the limbic system as an evolutionarily conserved “ancient” function is further reinforced by the presence of both steroid and enzyme in non-mammalian vertebrate reptiles (Callard et al., 1977).

The evolutionary conservation model fits within the evolution of “higher cognition” in mammals, such as humans. Humans express aromatase and estrogens in the limbic system, and frontal cortex where they play roles in cognitive ability (Sasano et al., 1998). As the human neocortex has develop, so too has our ability to utilize estrogens and the “Aromadition—the system under aromatase control” to alter structures depending on stress/learning interactions, exploiting a combination of rapid and sustained cellular mechanisms (Konkle and McCarthy, 2011; Saldanha et al., 2011; Srivastava et al., 2011). Furthermore, the discovery of neuroactive steroidal involvement in clinical investigation has highlighted a very real application of estrogens as pharmaceutical agents (Stoffel-Wagner, 2001).

Estrogens have shown the ability to enact change in a very short time scale. Through modulation of spinogenesis, synaptogenesis, and synaptic connectivity, estrogen is able to enact these rapid changes in neural circuits (Saldanha et al., 2011; Srivastava et al., 2013b; Sellers et al., 2015a,b). For examples, when rat cortical neurons were treated with 10 nM Estradiol, structural changes (spinogenesis) were observed within 30 min. Studies into the ability of estrogens to influence behavior also demonstrate a rapid influence of estradiol and estrogen receptor agonists (Srivastava et al., 2013b; Luine, 2014; Sellers et al., 2015a; Gobinath et al., 2017). However, these studies present a clear inverted U-shaped curve. Previous studies have suggested that either 1–2 or 5 μg/kg dose of estradiol receives a better effect (Inagaki et al., 2010), reflecting the optimal level of receptor dynamic activation (Srivastava et al., 2013b; Luine, 2014). However, this dose response may be limited to enhancement of behavioral and cognitive tasks.

Studies *in vivo* have shown that estrogen receptors have an integral function in the development of cerebral architecture. Specifically, the pyramidal cells of the hippocampus and the cortical laminae II-VI demonstrate expression of ER mRNA and ER protein (Shughrue and Merchenthaler, 2000). Studies in this area began to elucidate further evidence of the large scale of nuclear ERs spread across the cortex, particularly in laminae III-V (Shughrue et al., 1999). Evidence for the implication of estrogen receptors in organization of the cortex has been the discovery of extra-nuclear ERα in dendritic spines and astrocytes (Milner et al., 2000). ERß mRNA and protein has also been detected in the cortex of rats (Shughrue and Merchenthaler, 2001).

These data indicate that ERs are indeed expressed through the cortical laminae of rodents. However, much less is known about the expression of ERs and estrogen-related genes, transcription factors and proteins during development. Our preliminary studies examining the expression of aromatase has begun to illuminate potential pathways linking estrogen and development, particularly sexually dimorphic development.

Aromatase has been found to be highly expressed in pyramidal neurons as well as glial cells (Kretz et al., 2004; Yague et al., 2006, 2008). Consistent with this, there is increasing evidence that estradiol is produced within the neocortex even in the absence of sex organs in both male and female animals (Ish et al., 2007; Hojo et al., 2009). Critically, estradiol, as well as other steroids, have been measured in embryonic brains of male and female rats within the cortex (Konkle and McCarthy, 2011). These findings are mirrored by previous findings of aromatase activity in the cortex and hippocampus in perinatal animals (Tobet et al., 1985; MacLusky et al., 1994). These studies highlight the possibility that the *de novo* synthesis of estradiol, mediated by aromatase, represents a major source of estrogens within the brain (Cornil et al., 2006; Azcoitia et al., 2011; Saldanha et al., 2011; Srivastava et al., 2013b; Balthazart and Ball, 2017), and may influence the development of the cortex (Tobet et al., 1985; MacLusky et al., 1994; Konkle and McCarthy, 2011). It is also important to note that the three major estrogen receptors (ERs): ERα, ERβ, and G-protein coupled estrogen receptor 1 (GPER1), were found to be expressed in multiple brain regions, including the cortex (Mitra et al., 2003; Milner et al., 2005). Using the BrainSpan transcriptomic atlas of the developing brain (http://www.brainspan.org/), we found several of the key molecular players involved in estrogenic signaling are expressed in the developing human brain, namely *CY19A1* (aromatase), ESR1 (ERα), ESR2 (ERβ), and GPER1 (GPER1). Specifically, these genes were found to be expressed during the developmental period spanning embryonic to late prenatal in the dorsal frontal cortex (DFC), ventral frontal cortex (VFC), and the hippocampus (HIP). CYP19A1 expression was found to increase throughout development, within these regions. Similarly, ESR1 trends toward an increase during over development, whereas ESR2 seems to be highly expressed during early corticogenesis before decreasing slightly then plateauing until late prenatal stage (Gatford, Denley and Srivastava, unpublished observations). Interestingly, the recently identified estrogen sensitive G-protein coupled receptor, GPER1 (also known as GPR30) (Srivastava and Evans, 2013) increases in its expression throughout corticogenesis. Combined, these data support the notion that the molecular machinery required for estrogenic signaling is expressed throughout early development within the cortex, and further suggests that estrogens may play a role during corticogenesis. Consistent with these data, we find that aromatase is expressed in multiple cell types within the brain of post-natal day 0 mice (Bakker et al., 2002; Karolczak et al., 2008). It should be noted that during brain development, estradiol has an organizational role and is central to the sexually dimorphic development and regulation of the neural circuitry underlying sex-typical and socio-aggressive behaviors in males and females (Nelson and Trainor, 2007; McCarthy and Arnold, 2011; Ubuka and Tsutsui, 2014). Interestingly, these effects seem to be in part mediated by the active repression of DNA methylation in the POA and hypothalamic ventromedial nucleus (Nugent et al., 2015; Mosley et al., 2017). Ultimately, whether such mechanisms are also important for the development of cortical circuitry is currently unknown. Nevertheless, taken together, these data suggest that estrogens, and aromatase, are not only present, but also actively regulated during key phases of the developing cerebral cortex.

Another line of evidence that estrogens play an important role in the development of the cortex in a sex specific manner comes from studies examining the detrimental effects of Bisphenol A (BPA). BPA is a Xenobiotic^*^ and antagonist to estrogens via ERs. It has been noted that BPA alters sexual differentiation in early development. Males, but not females, exposed to BPA at 400 μg/kg altered the number of neurons and glial cells in the deep layers of the medial prefrontal cortex (Sadowski et al., 2014). Altered expression of ERs, which has shown to be sex-specific may account for this biased and unexpected mechanism (Kundakovic et al., 2013). It should be noted that BPA also binds androgen receptors (Kuiper et al., 1998; Sohoni and Sumpter, 1998), which may confer some of these effects. Sexually dimorphic neuron progeny may be attributed to the divisive effects of BPA among sexes, as specific neurons comprising sexually dimorphic regions are produced at markedly different timespans (Jacobson and Gorski, 1981). Nevertheless, the effects of BPA on neuronal and glial cell volumes within the cortex (Kubo et al., 2003) provides a strong case for estrogenic signaling in cortical development, and suggests that the steroid hormone may account for some sexual dimorphism seen in this area. However, one additional caveat of using BPA as an example to demonstrate a role for estrogens during corticogenesis, is its universal effects on other tissues in the body. The thyroid is one such example; environmental contamination by BPA results in up-regulation of thyroid hormone-responsive genes in the dentate gyrus (Zoeller et al., 2005), which is an area that has shown sexual dimorphic characteristics (Roof, 1993; Tabibnia et al., 1999). As highlighted above, BPA also strongly inhibits the activity of androgen receptors (Sohoni and Sumpter, 1998), which will have a considerable impact on the development and function of the cerebral cortex (Clark et al., 1988; Nuñez et al., 2003). A more comprehensive summary of BPAs effects can be found in Wetherill et al. (2007).

Maternal consumption and/or exposure to xenobiotic compounds can enter into the amniotic environment of the fetus and effect neurodevelopment. Exposure to xenobiotic compounds at physiological levels and from the environment can be detected in the amniotic fluid, which introduces it to the fetus (Nikaido et al., 2004; Engel et al., 2006). An area of increasing concern is that regarding dietary phytoestrogens. This area also provides further evidence to support the argument of estrogen and aromatase as key regulators of cortical development. In numerous animal models, manipulation of estrogen function by dietary phytoestrogens during gestation can lead to disrupted brain organization (Gorski, 1985; Lindzey and Korach, 1997). It has been suggested that dietary phytoestrogens are easily diffusible across the placenta and may then interfere with development (Patisaul and Jefferson, 2010). One must be cautious to draw conclusions from this, as absorption and concentration may differ wildly, especially, considering other dietary factors. In rodent studies, phytoestrogens such as polyphenols, flavonoids, and isoflavonoids have been shown to readily cross into the placenta and into the fetus brain (Doerge et al., 2001). Furthermore, during the prenatal period, systemic circulation of phytoestrogens is considerably more efficient compared to adults (Chang et al., 2000). Three anatomical locations have been particularly prominent in researching estrogen and epigenetics, the preoptic area (POA), locus coeruleus (LC), and the hypothalamic-pituitary axis (HPA), specifically the ventromedial nucleus. The POA and LC show sexual dimorphic areas, with ERα playing the more dominant role over ERß in both cases (Shughrue et al., 1997; Pérez et al., 2003; Patchev et al., 2004). Yet this raises the question, whether there are sexual dimorphic areas, hitherto undiscovered, that ERß maintains dominance?

The rodent POA has shown to be heavily sensitive to estradiol aromatized from gonadal androgens (Gorski, 1985). This is reflected in the greater volume of the male POA in comparison to the female POA. Furthermore, the expression of aromatase is variable not just in areas of sexual dimorphism, but also in response to testosterone and estrogen exposure (Roselli and Stormshak, 2010). Within the POA, the phytoestrogen genistein acts as an agonist to estrogen receptors. Exposure to genistein results in increased volumes in male but not female calbindin labeled sexually dimorphic areas (Scallet et al., 2004). This particular example highlights the importance of androgen synthesis and pharmacological dynamics, which are unfortunately beyond the scope of this literature review. Conversely, in the pre- and postnatal LC, exposure to the phytoestrogen resveratrol demasculinizes the male brain resulting in sexual dimorphism to volume and cell density (Kubo et al., 2003). It is pertinent to add that androgens are chiefly responsible for masculinizing the male brain. However, it would be irresponsible to not give weight to the estrogen (perhaps more so of ERα) mediated changes in organizational structure of the brain, with this example showing the ability of estrogen and aromatase to change both cerebral volume and cell densities in discrete areas.

## Cortical development

Episodic processes of progenitor proliferation, neural differentiation, polarization, neuronal migration and lamination underpin the cortical development pattern (Ohtaka-Maruyama and Okado, 2015). Through selective expression of transcription factors and interaction with extra-cellular protein receptors, cortical layers are formed “inside-outside” (Figure 1; Angevine Jun and Sidman, 1961). Late-borne progenitors will ascend to the pial layers, whereas early-borne progenitors remain in deeper layers, which is modulated and regulated through transcription at the various episodes. Below, we highlight some of the key cell types and events that are critical for normal corticogenesis.

### Proliferation and progenitor maintenance

**Figure 2:**
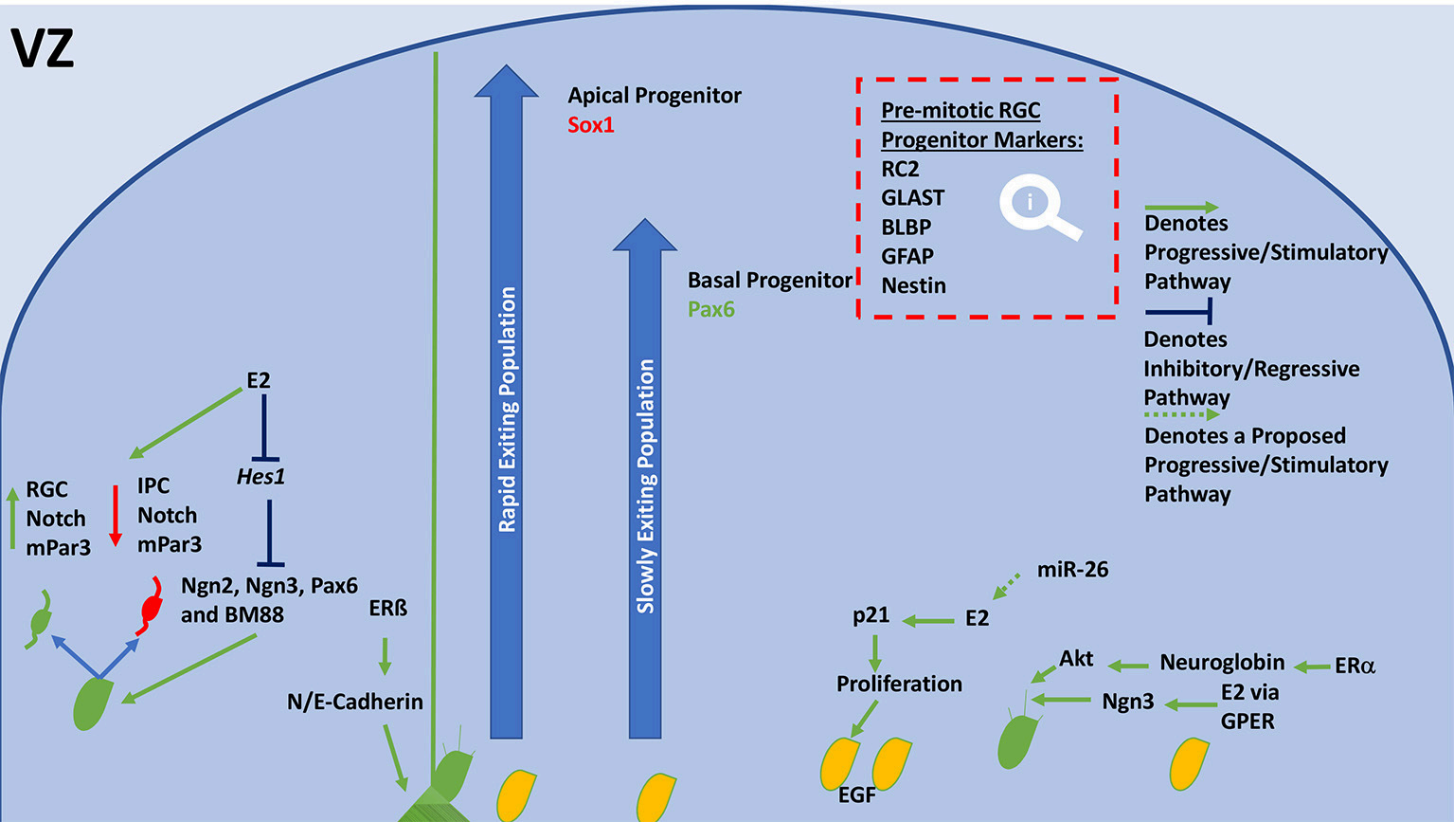
General schematic diagram of molecular events that regulate neurogenesis and proliferation in the VZ. Neuroblasts, or NSCs divide asymmetrically, their progeny inherits either high notch and mPar3 or low notch and mPar3. RGCs receive higher notch signaling, their counterpart receives lower notch signaling and becomes an IPC. E2 inhibits Hes1 expression, resulting in increased division by inhibition of neurogenins, Pax6, and BM88. ERß is able to modulate N and E-Cadherin levels, which stabilizes end-feet of radial scaffolds and mediates adhesion. Depending on the expression of Sox1 or Pax6 a progenitor will join the rapidly or slowly ascending pool. Sox1-expressing populations (Apical progenitors) are released rapidly but remaining in the deeper layers of the CP. Pax6-expressing populations (Basal progenitors) are released slowly and comprise the superficial neurons of the CP. E2 has also shown to increase proliferation of NSCs through stimulation of p21 and increasing EGF expression. miR-26 may increase E2 synthesis or ER expression to meet this end. E2 is also able to increase neuritogenesis, leading to the formation of an axon. It accomplishes this via GPER and ERα, which are able to stimulate neuritogenesis through Ngn3 and Neuroglobin/Akt, respectively. E2, Estradiol; VZ, ventricular zone; RGC, radial glial cell; NSCs, neural stem cells; IPC, intermediate progenitor cell; Ngn, neurogenin; GLAST, astrocyte-specific glutamate transporter; BLBP, brain-lipid binding protein; GFAP, glial fibrillary acidic protein; ERα, estrogen receptor alpha; ERß, estrogen receptor beta; GPER, G-protein estrogen receptor; EGF, epidermal growth factor; CP, cortical plate.

**Figure 3:**
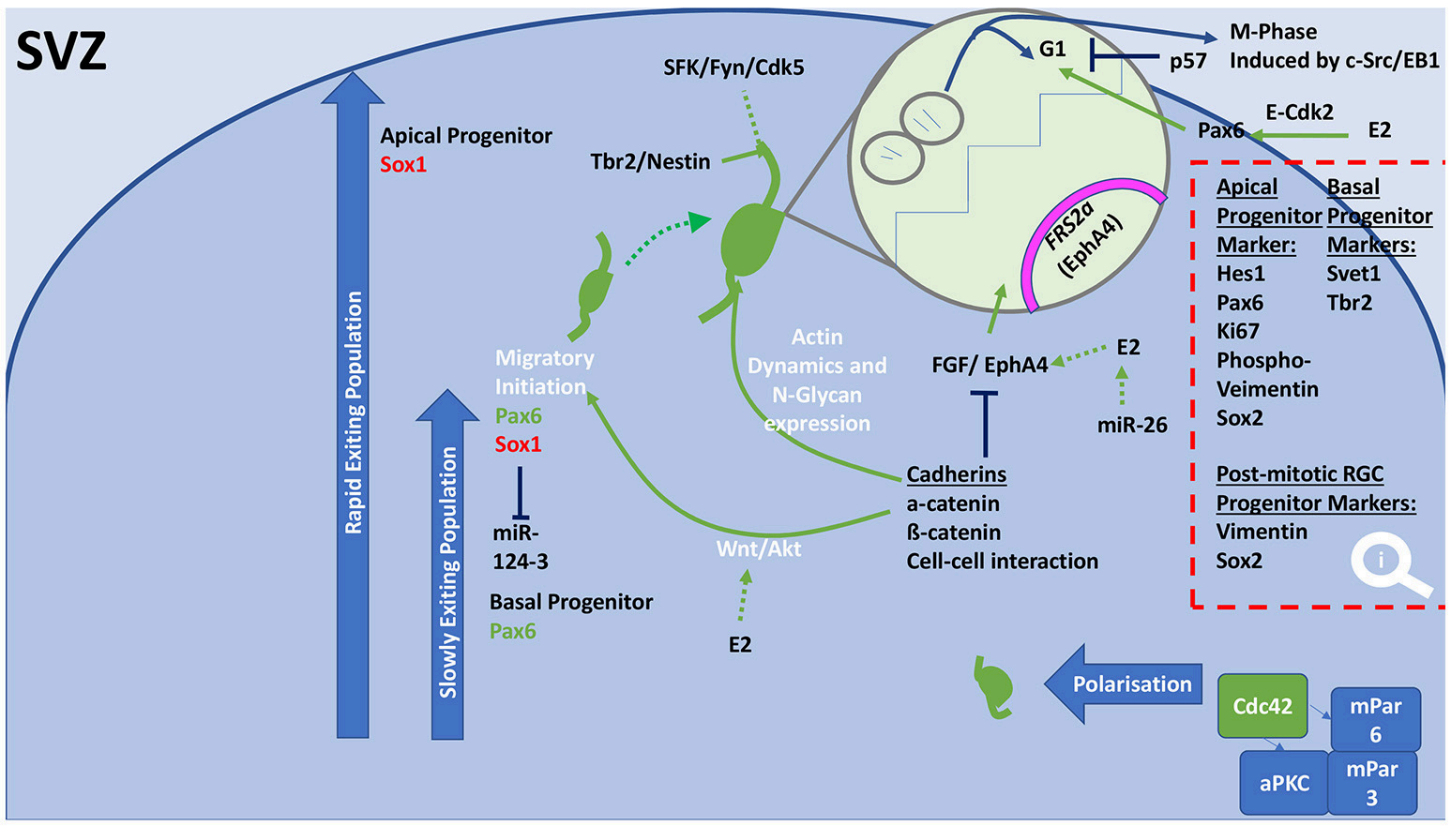
General schematic diagram of molecular events that regulate proliferation, differentiation and the initiation of migration in the SVZ. Progenitor populations in the SVZ and outer SVZ undergo proliferation, differentiation, polarization, which are controlled to achieve either neural population increases or migration. In both these populations, collections of cellular processes are regulated to shift balance between proliferation and differentiation. Cadherins cause cell-cell interactions through Wnt/Akt signaling, which result in altered levels of Pax6 or Sox1 expression. Cadherins increase expression of actin dynamic molecules and N-glycan. Resulting in axonal growth and migration, which involves incorporation of Tbr2, Nestin, and the SFK/Fyn/Cdk5 pathway. Cadherins influence the cell cycle through inhibition of the growth factor FGF and EphA4, which results in decreased proliferation and increased differentiation to allow migration. E2 is able to increase FGF/EphA4 action leading to greater proliferation. This action is achieved through E2 stimulating Pax6 through E-Cdk2. Polarization of migrating neurons in the SVZ can be accomplished by the coupling of Cdc42 to an aPKC/mPar6/mPar3 complex. SVZ, subventricular zone; miR, microRNA; E2, Estradiol; ERα, estrogen receptor alpha; ERß, estrogen receptor beta; GPER, G-protein estrogen receptor; aPKC, atypical protein kinase C; FGF, fibroblast growth factor; FRS2a, fibroblast growth factor receptor substrate 2a; G1, Gap 1 phase; SFK, Src family kinase; EphA4, Ephrin type-A receptor 4; Fyn, Src tyrosine-protein kinase fyn; NSCs, neural stem cells.

In proliferative and neurogenic states during development, progenitors are produced through division and proliferation of neural stem cells (NSCs) in the ventricular zone (VZ) (Figure 2) and sub-ventricular zone (SVZ) (Figure 3). Basal progenitors of the SVZ, identified by the expression of Svet1 and Tbr2, of the SVZ undergo symmetrical division and produce two neurons. Vimentin and Sox2 staining can highlight radial glial cell (RGC) (Figure 2) progenitors of the outer SVZ (Lancaster et al., 2013). Ngn3 contributes to maintaining progenitor oligodendrocytes in the SVZ (Ivanova et al., 2003) (Figure 3). Only progenitors of the VZ expressed Par complex rich domains, which develop into apical progenitors (express: Hes1, Pax6, Ki67, Phospho-vimentin, and Sox2; Knoblich, 2008) (Figures 2, 3). RC2, GLAST, BLBP, Nestin, and GFAP staining can also highlight RGC progenitors of the VZ (Rakic, 1971; Hartfuss et al., 2001; Liu et al., 2017; Figure 2). Hes1 gene transcription regulates VZ progenitor pools and proliferation. However, RGCs typically divide asymmetrically, and thus, increase the diversity of daughter cells (Noctor et al., 2004). However, during highly proliferative episodes, symmetrical division is favored to increase the progenitor supply pool. Mechanistically, this process is mediated through Wnt signaling, leading to an increase in progenitor pool (ß-catenin-dependent) (Galceran et al., 2000). Interestingly, Par inheritance is directly linked to inherited Notch signaling capability (Kon et al., 2017; Bultje et al., 2018), furthermore, Notch signaling is required for neurogenesis and the maintenance of VZ NSCs (Imayoshi et al., 2010). Progenitors in the SVZ inherit or express high levels of Cux1 and Cux2, which signals them as apical progenitors (Nieto et al., 2004; Figure 3). G1 phase mitosis is a key regulatory point in proliferation (Dehay and Kennedy, 2007). CDK inhibitors, p27 and p57 coordinate corticogenesis and cell cycle exit. Prior to differentiation, p57 is responsible for cell cycling (Bilodeau et al., 2009). BM88 influences NSCs to exit the cell cycle and is expressed by RGCs (Koutmani et al., 2004).

Asymmetric inheritance is also seen in Pax6 and Ngn2 positive RGCs (Kawaguchi et al., 2004). Pax6 regulates neurogenesis and proliferation in the VZ and SVZ (Gan et al., 2014) and maintains the SVZ progenitor pool (Wong et al., 2015). Pax6 activity has shown to be ß-catenin-dependent. A dynamic relationship between Sox1 and Pax6 controls transgression through periodical cortical development states (Suter et al., 2009; Wong et al., 2015). EphA4 binds to fibroblast growth factor (FGF), which is a potent stimulator of cortical proliferation (Collette et al., 2017). EphA4 regulates FRS2α (Yokote et al., 2005), which activates downstream RAS-MAPK and PI3K signaling pathways (Ornitz and Itoh, 2015).

### Differentiation and polarization

Differentiation of progenitors causes morphological changes, which allows migration and maturation. Differentiation and migration require dynamic cytoskeleton changes; extracellular and intrinsic signaling tightly regulates these events. For example, cadherins alter cellular dynamics (Nagafuchi et al., 1987). N-glycan-dependent attachment of cells recruits cadherin, causing greater cell-cell interaction (Hall et al., 2014). Nestin signals morphological changes within RGCs localized to axonal projections extending from the VZ (Xu et al., 2015; Vinci et al., 2016). Tbr2 generates transcriptional changes to cause cell to select a leading (axonal) strand (Sessa et al., 2008) (Figure 2). Ngn2 is downregulated by Hes1, which shifts a cell toward differentiation by modulating Notch signaling (Niwa et al., 2009; Figures 2, 3). Ngn3 is upregulated to stimulate dendritogenesis and synaptogenesis. CRM1 mediates the nucleo-cytoplasmic shuttling of Ngn3, allowing transcriptional change (Simon-Areces et al., 2013) (Figures 2, 3). In addition to its effect on proliferation, Wnt signaling utilizes ß-catenin-dependent pathways to differentiate intermediate progenitors into neurons (Clevers, 2018). Interestingly, cadherins also employ this pathway to mediate cell cycling (Zhang et al., 2013). Indeed, cadherins activate Wnt and Akt to switch from proliferation to migration (Ajioka and Nakajima, 2005).

**Figure 4:**
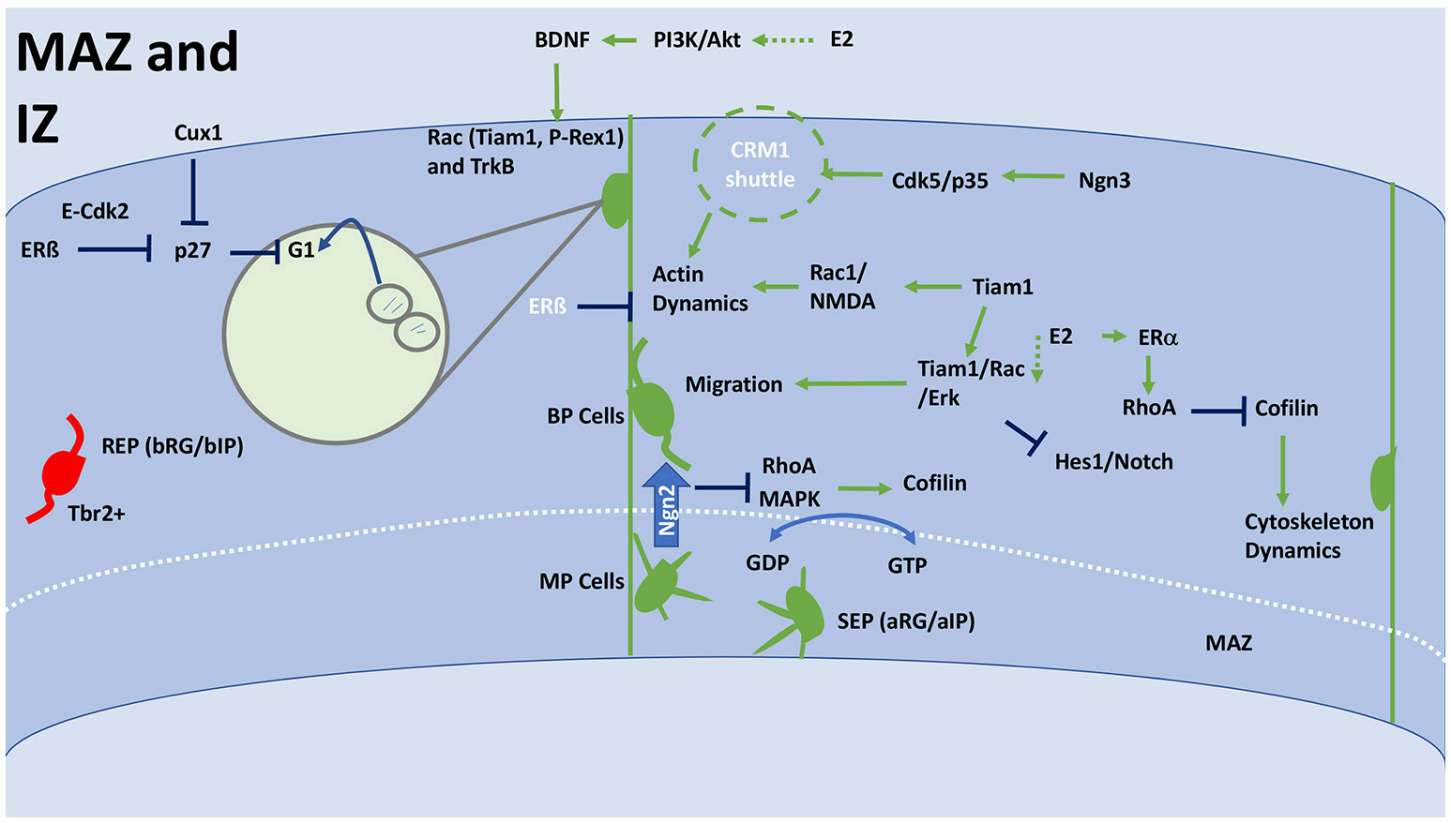
General schematic of corticogenesis in the IZ and MAZ, including differentiation and migration. The IZ facilitates cytoskeleton changes that affect the morphology of the migrating cell, much of this will occur in the MAZ. The MAZ is an accumulation ground for multipolar cells that are forming into mature bipolar neurons, which can then enter into the CP. The MAZ is comprised of two populations SEP and REP. Whereas the REP population passes straight through the MAZ, the SEP remains in the MAZ for much longer. Ultimately, the SEP forms the more superficial layer of the CP. REP can be distinguished from SEPs as they are Tbr2+. Crossing from the MAZ to IZ requires a transition from MP to BP, which is initiated by Ngn2, which inhibits RhoA and MAPK that have a downstream effect on Cofilin. Cofilin interacts with RGCs to increase cytoskeleton dynamics and affect a neuronal morphology. Cofilin can also be inhibited by RhoA, which is modulated through ERα. Oscillation between GDP and GTP control orientation and polarization of cells within the MAZ. This is achieved through lamellipodium formation and interaction with the cofilin system. Upregulation of Tiam1 or E2 can result in activation of Tiam1/Rac/Erk signaling, which stimulates BP cells to migrate through the IZ. Tiam1 also activates Rac1/NMDA to alter actin dynamics, which results in increased elongation, synaptogenesis and dendritogenesis. This process can be negatively regulated by ERβ or actively upregulated by Ngn3 through Cdk5/p35 activating a CRM1 shuttle. RGCs are stimulated to move toward the CP by interaction with BDNF. E2 stimulation via PI3K/Akt signaling can cause BDNF expression. BDNF binds to TrkB and upregulates Rac/Tiam1/P-Rex1 activity. Through Cux1 and E2 (ERβ), proliferation is inhibited at this stage allowing for migration to take place. Both Cux1 and E2 inhibit p27, which stops proliferating cells at the G1 phase. E2, estradiol; IZ, intermediate zone; MAZ multipolar cell accumulation zone; CP, cortical plate; RGC, Radial glial cell; ERα, estrogen receptor alpha; ERβ, estrogen receptor beta; BP, bipolar; MP, multipolar; SEP, slow exiting population; REP, rapidly exiting population; BDNF, brain-derived neurotrophic factor; NMDA, N-methyl-D-aspartate; TrkB, Tropomyosin receptor kinase B; MAPK, mitogen-activated protein kinase; PI3K, Phosphoinositide 3 kinase; Akt, Protein Kinase B; bRG/bIP, basal radial glia/intermediate progenitor; aRG/aIP, apical radial glia/intermediate progenitor; G(D/T)P, guanosine (di/tri)-phosphate; CRM1, chromosomal maintenance 1/exportin 1; Cux1, cut like homeobox 1; RhoA, Ras homolog gene family, A; EphA4, Ephrin type-A receptor 4; Tiam1, T-cell lymphoma Invasion And Metastasis 1; Erk, extracellular signal-regulated kinases; Tbr2, T-box brain 2; E-Cdk2, G1 phase specific Cyclin E, Cyclin-dependent kinase 2; NSCs, neural stem cells.

Switching mechanisms and alterations in morphology are crucial to poising the cell to undergo polarization. The sub-plate of the IZ is a multipolar cell accumulation zone (MAZ) (Figure 4). Beyond the MAZ cells are mostly bipolar or unipolar neurons. Ngn2 regulates the transition of polarity (Ohtaka-Maruyama and Okado, 2015). Ngn2 inhibits RhoA, causing a cascade that downregulates MAPK (Zeidan et al., 2014) (Figure 4). RP58 regulates Ngn2-mediated morphological changes (Ohtaka-Maruyama et al., 2018). Interaction between ABP/cofilin/myosin inhibits RhoA through Rnd3 causing filopodial and lamellipodial growth and retraction (Pacary et al., 2011; Gomez and Letourneau, 2014). GDP-bound and GTP-bound oscillation regulates lamellipodium formation that orientates cells within the MAZ (Sailland et al., 2014). Par complex proteins (Par3, Par6, aPKC) cause apical base neurons to undergo polarization through Cdc42 coupling (Cappello et al., 2006). A compressive review of polarization can be found in Polleux and Snider's paper Polleux and Snider.

### Migration and lamination

Glial-guided neurons use cellular locomotion to migrate to the CP. Once in position, neurons attach their leading strand to the marginal zone (MZ) and switch to somal translocation. Chemotaxis is mediated through membrane-bound receptors and extracellular matrix proteins, including Rac, brain-derived neurotropic factor (BDNF) and TrkB signaling (Figure 4; Zhou et al., 2018). Calcium-dependent mechanisms recruit BDNF (Zhou et al., 2018). BDNF binding to TrkB stimulates axonal growth and dendritic morphological change (Cohen-Cory and Fraser, 1995; Wang et al., 2015) (Figure 4). The selection and elongation of a leading (axonal) strand stimulates migration. Migration can be induced by Tiam1/Rac1/ERK signaling (Xiao et al., 2013). Interaction between the SFK Fyn and serine/threonine kinase family member Cdk5 are evidenced to play a leading role in progenitor cell axon guidance and dendritic orientation (Sasaki et al., 2002; Figure 4). Reelin and Dab1 alter cytoskeleton morphology through CLASP2 (Dillon et al., 2017). Cux1 and Cux2 regulate dendritic morphology through Pax6 and p27 in pyramidal neurons (Nieto et al., 2004).

**Figure 5:**
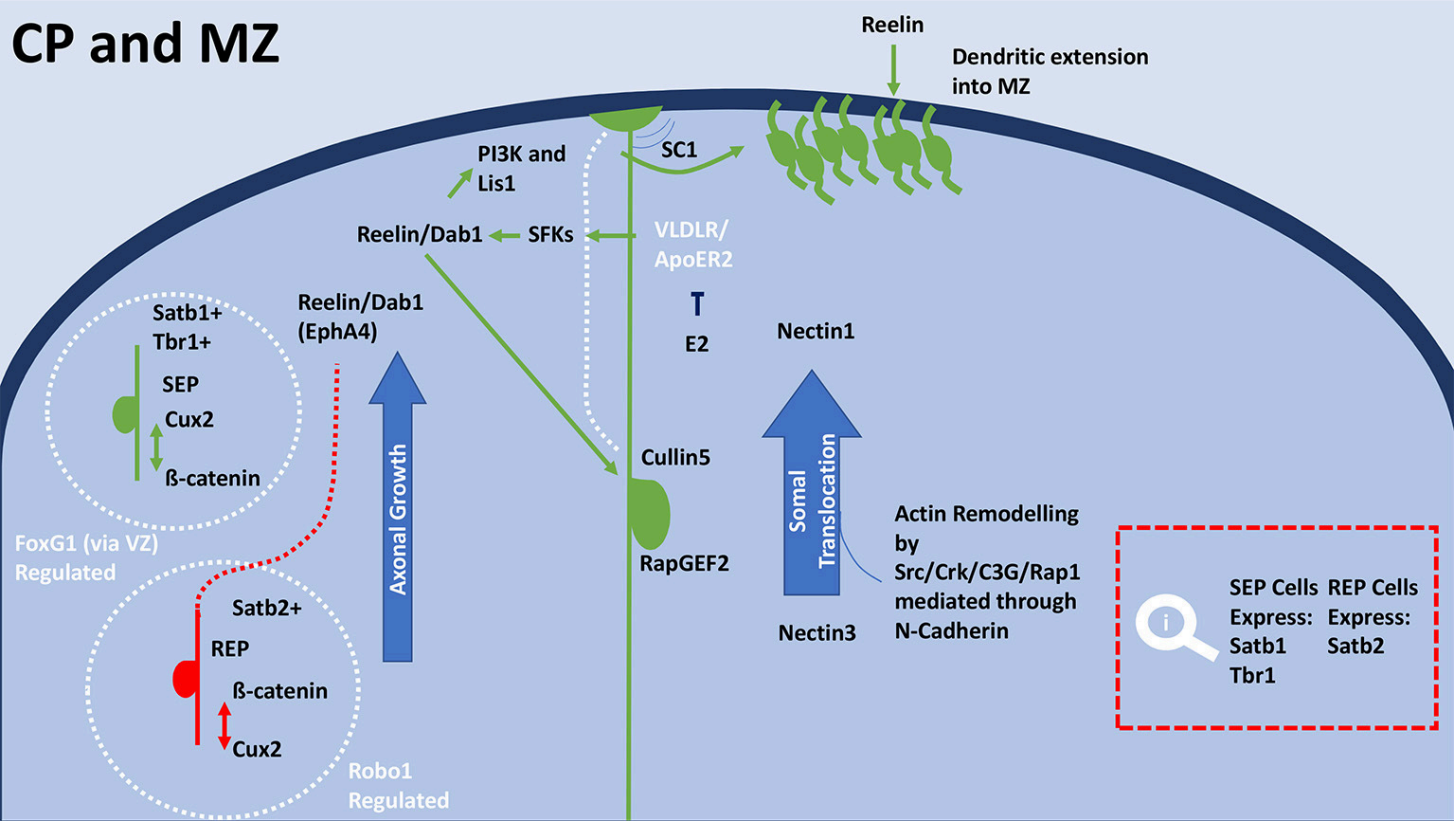
General schematic of terminal translocation and differentiation in the CP during development. Within the CP SEP neurons (green) have high expression of Cux2 and low expression of β-catenin, whereas REP neurons (red) have high expression of β-catenin and low expression of Cux2. Satb1 positive cells express in the superficial layers of the MZ, which overlap with Tbr1 expression. Terminal translocation and therefore lamination rely on regulation by FoxG1 for the SEP neurons and radial distribution of Satb2 expressing REP neurons is regulated by Robo1. FoxG1 on deep-layer progenitors through transcription switches their progeny to upper-layer neurons through repression of Tbr1. After exiting the cell cycle, Satb2-expressing cells immediately migrate to the upper layers of the cortical plate. Satb2 expressing cells are much more reliant on the reelin/Dab1 and Ephrin-A pathways for cortical migration. Reelin binds to the RGCs by VLDLR and Apoer2 receptors (Lane-Donovan and Herz, 2017), which causes the adaptor protein Dab1 to become phosphorylated. Upon phosphorylation by SFKs, Dab1 recruits various downstream molecules including PI3K and Lis1. E2 is able to inhibit VLDLR/ApoER, modulates reelin's mechanisms in cortical migration. Reelin's interaction with cadherin is also essential for the termination of migration. Through regulating terminal translocation, the reelin/Dab1/Rap1/N-Cadherin signaling pathway leads to the inside-out lamination of the cortex. Nectin molecules expressed in the Cajal-Retzius cell (Nectin1) and the migrating neuron (Nectin3) are also necessary for somal translocation. The initiation of detachment is signaled by SC1, which is expressed on the surface at the top and bottom of RGCs surfaces. The anti-adhesive signal is crucial to proper cortical development, as the absence of SC1 results in failure of neurons to detach and properly position. Dab1 interacts with Cullin5 in the migrating cell to accumulate in the appropriate cortical layer. Termination of polarization upon reaching the appropriate location is met by the degradation of reelin receptors, N-cadherin and Dab1 by exocytosis and endocytosis. E2, Estradiol; IZ, intermediate zone; MAZ, multipolar cell accumulation zone; CP, cortical plate; RGC, Radial glial cell; ERα, estrogen receptor alpha; ERβ, estrogen receptor beta; BP, bipolar; MP, multipolar; SEP, slow exiting population; REP, rapidly exiting population; SFK, Src family kinases; PI3K, Phosphoinositide 3 kinase; SC1, SPARC-like1; VLDLR, very low-density lipoprotein receptor; ApoER2, apolipoprotein E receptor 2; Cux2, cut like homeobox 2; FoxG1, Forkhead Box G1; Dab1, Disabled-1; Tbr1, T-box brain 1; Lis-1, Lissencephaly-1; Crk, (p38/adaptor molecule crk); C3G, CRK SH3-binding GNRP; Rap1, Ras-like GTPase; Satb(1/2) Special At-rich sequence binding protein (1/2); Robo1, Roundabout Guidance Receptor 1; EphA4, Ephrin type-A receptor 4; NSCs, neural stem cells.

The lamination process relies on signaling pathways that halt migration and trigger neuronal maturation through terminal and somal translocation. Reelin, Dab1, Wnt/Fizzled, and SPARC-like1 (SC1) are essential for this process. Wnt/Frizzled and reelin signaling regulates boundary formation during lamination (Augustine et al., 2001; Brault et al., 2001; Franco et al., 2018). SPARC-like1 (SC1) signals detachment of RGCs from the membrane. Terminal translocation in neurons is signaled by Dab1 and Cullin5 interaction (Feng et al., 2007; Figure 5). Migrating neurons interact with reelin through RapGEF2 (Ye et al., 2014). Neurons extend dendrites into the CP, which requires elongator to acetylate α-tubulin resulting in microtubule reassembly (Creppe et al., 2009; Heng et al., 2010). Interaction between reelin and VLDLR and ApoER2 causes Dab1 phosphorylation, which recruits PI3K and Lis1 (Bock et al., 2003; Chai and Frotscher, 2016; Lane-Donovan and Herz, 2017). Nectin1 and Nectin3 show capability of facilitating and mediating somal translocation (Hirota and Nakajima, 2017; Figure 5). N-cadherin interacts through the Nectin's cytoplasmic domain region.

## Cytoarchitecture-specific mechanism involved in sexual differentiation

**Figure 6:**
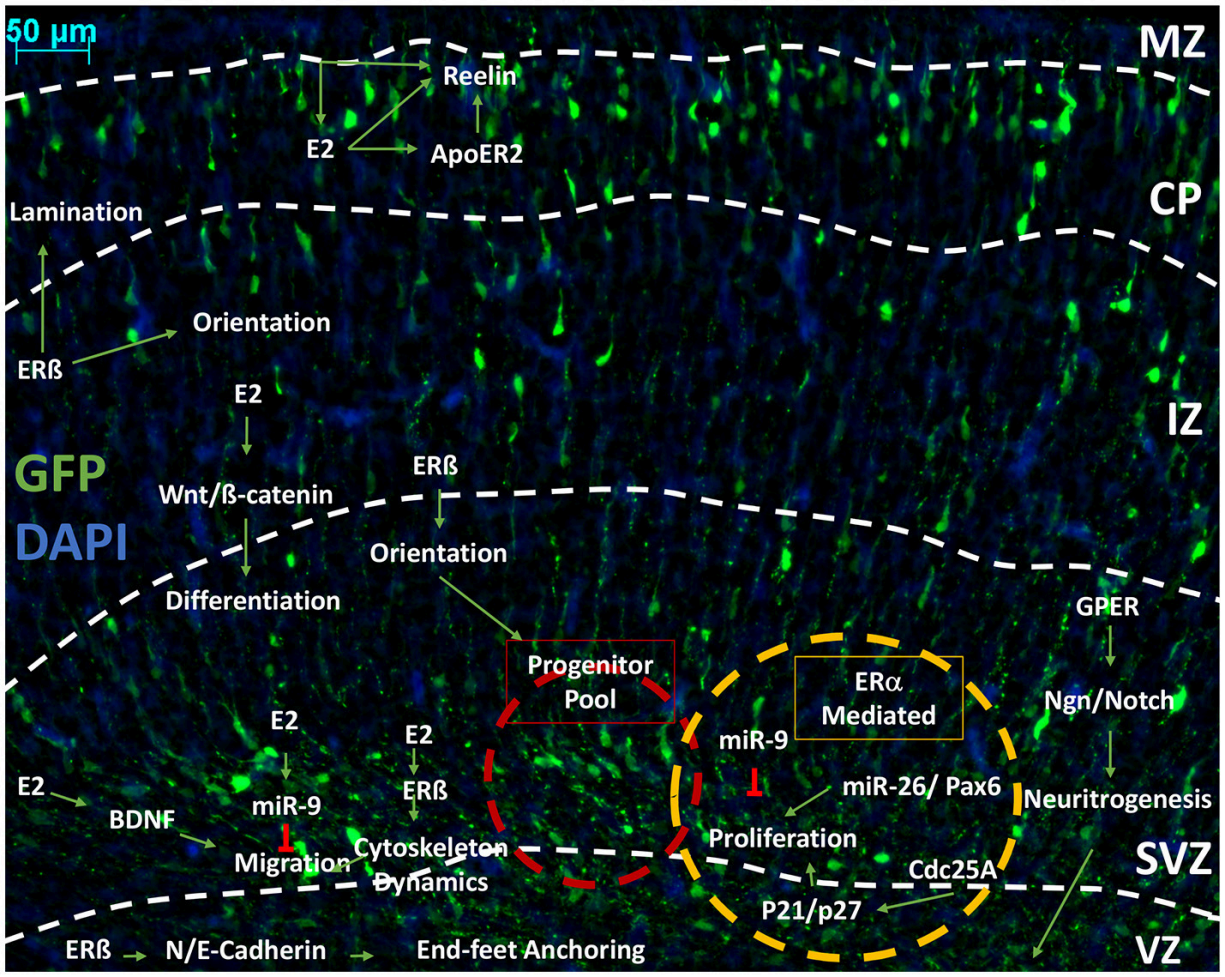
An outline of the pathways influenced by estrogenic signaling to drive cortical development. Representative image of developing cortex. Electroporation of eGFP was performed at E14.5 and brains collected at P0 as previously described (Srivastava et al., 2012a,b). Representative image of a coronal rodent brain slice at P0 during cortical development. Estrogen (Estradiol/17ß-estradiol) is shown interacting in pathways outlined in Figures 1–4. that are involved in neurogenesis, proliferation, differentiation and migration. We propose that Estradiol may be able to affect the proliferative status of the progenitor pool through Pax6 and kinase inhibitors through ERß or ER. Through ERß, Estradiol can affect migration by marking terminal translocation points, therefore setting lamination boundaries. Estradiol is able to influence orientation through a number of mechanisms, which has been shown specifically in GFAP+ RGCs and mediated through ERß. Through ERß, Estradiol also alters the cytoskeleton dynamics to initiate migration. Cytoskeleton dynamics are also affected by GPER to increase neuritogenesis, which will later form axons and dendrites. Estradiol also alters differentiation state by increasing Wnt/ß-catenin signaling, which furthers dendritogenesis. Estradiol is able to negatively regulate migration and proliferation by increasing miR-9, which inhibits these processes. In regards to proliferation, it is hypothesized that ERα is primarily responsible for mediating the action of estrogenic signaling. Estradiol also interacts with reelin signaling in the CP and MZ, both directly by increasing expression and indirectly by stimulation of ApoE gene expression, which increases the activity of ApoER to affect the morphology of the cortex and cytoarchitecture of the CP. E2, estradiol; IZ, intermediate zone; MAZ, multipolar cell accumulation zone; CP, cortical plate; ERß, estrogen receptor beta; ERα, estrogen receptor alpha; GPER, G-protein estrogen receptor; miR-x, microRNA; ApoER2, apolipoprotein E receptor 2; Wnt, Wingless-type; BDNF, brain-derived neurotropic factor; p21, cyclin-dependent kinase inhibitor 1A/Cip1; p27, cyclin-dependent kinase inhibitor 1B/Kip1; Ngn, neurogenin; Cdc25A, Cell division cycle 25 homolog A; VZ, ventricular zone; SVZ, subventricular zone.

Sexual differentiation in the central nervous systems occurs throughout vertebrate evolution, including human beings (Jazin and Cahill, 2010). Hypothalamic nuclei are profoundly sensitive to sex-dependent change. The anteroventral periventricular (AVPV) nuclei in the hypothalamus is typical of the sexual dimorphism. Due to caspase-dependent apoptotic signaling induced by perinatal exposure to estradiol (Waters and Simerly, 2009), males typically have a smaller AVPV than their female peers (Simerly et al., 1985). The phenotype of this is a decreased cellular volume (Forger et al., 2004). Estradiol has shown to be a crucial hormone in regulating and mediating the development of the neocortex (Beyer, 1999; McEwen and Alves, 1999). For example, ERß knockout mice (ERβKO) have revealed that ERβ is necessary for cortical lamination, which indicates a potential role for estradiol in orientation through actin dynamics (Wang et al., 2003), although the mechanisms by which this occurs is poorly understood. Ultimately, it is not clear if this mechanism may lead to sexually dimorphic phenotypes. A number of studies have shown that estradiol increases the proliferative status in embryonic NSCs (Fried et al., 2004), which involves ERs but is limited to neurogenesis in embryonic development (Brännvall et al., 2002). Estradiol-induced proliferation of NSCs is mediated through the classic estradiol receptors, ERα and ERβ (Okada et al., 2010). Hitherto, the literature has not provided a comprehensive summary of the finer biochemical interactions of estradiol, which can be seen in many pathways mentioned above and throughout the cortex. In the proceeding sections, we review the evidence that indicates specific molecular players involved in corticogenesis and how these may be regulated in a sexually dimorphic manner. In addition, data that link estradiol-signaling with the regulation of key molecular players involved in the distinct and carefully orchestrated events described in the previous sections (Figure 6).

### Markers of proliferation in sexual dimorphic areas

Sexual dimorphism during development can be attributed to physiological factors such as hormonal exposure, as well as cell-intrinsic mechanisms that lead to differentiation (Swaab et al., 1995; Tsukahara, 2009). Cell-specific differentiation can be met through numerous factors, some of which will be discussed here.

The cellular state of neural progenitors can provide a comprehensive depiction on the condition of neurogenesis. As a marker of proliferation Ki67 expression in the discrete developing areas, can provide evidence to draw conclusions about total cell volumes at maturity. Infant rat preoptic area and hypothalamus show higher expression of Ki67, when compared to adults (He et al., 2013). Furthermore, colocalisation of Ki67+ cells and estradiol is noted in the preoptic area and hypothalamus.

Nestin has also been implicated in generating larger cells volumes in sexually dimorphic ventricular areas, such as the POA (He et al., 2013). Estradiol treatment on rat telencephalon cultures did not have an effect on the proportion of nestin-positive cells (Okada et al., 2010). However, estradiol did affect the cell fate of progenitor cells from the telencephalon of rats, which caused an increase in the ratio of oligodendrocytes being produced (Okada et al., 2008). The rostral third ventricle is the site of stem cell niches that strongly express nestin, which supplies the frontal and medial neocortex with neuroblasts (He et al., 2013). Estradiol has significantly strong proliferation-promoting effects in the 3rd ventricle stem cell niche (He et al., 2015). The effect of estradiol on nestin-expressing stem cell niches may serve to further reinforce the proliferative proprieties of estradiol in the developing cortex.

Ki67 and nestin expression colocalized with estradiol in sexually dimorphic areas is suggestive of a possible mechanism. The inclusion of a proliferative marker and intermediate filament protein is suggestive that estradiol increases the proliferative state and leads to greater migration through radial axon growth (Scholzen and Gerdes, 2000; Xu et al., 2015).

### Glial fibrillary acidic protein (GFAP)

GFAP is a robust astrocytic marker of RGC progeny (Berman et al., 1997). Immunoreactive GFAP has been detected in neural circuits that display sexual dimorphism. These circuitries can be manipulated through introduction of exogenous steroid hormones (Martinez et al., 2006). In studies primarily focusing on the cortex, ovariectomised rats showed higher rates of cortical proliferation resulting from treatment with estradiol (Malinowska-Kolodziej et al., 2009). The study also noted co-localization of nestin and GFAP in the cortex, which can be suggested as a mark of increased radial glial migration resulting from estradiol treatment. The substantia nigra of ovariectomised parkinsonian model mice did not respond to estradiol in the same manner, with GFAP remaining unchanged (Yi et al., 2016). However, Yi et al.'s team used BDNF as a marker to show an estradiol-dependent increase in net migration of neurons in the midbrain. These results suggest that estradiol within the cerebral cortex is able to increase proliferative status. The interaction between GFAP and estradiol is likely mediated through ERß. Indeed, Zsarnovszky et al. (2002) showed that estradiol via ERß is able to directly regulate GFAP expression in the interpeduncular nucleus, an important component of the limbic midbrain circuitry. Furthermore, agonists of ERß, namely LY3201, caused immunoreactive GFAP in hippocampal and cortical astrocytes to increase (Tan et al., 2012). This study also found that dendritic spines could be reduced using an ERß agonist (Tan et al., 2012). This suggests the mechanism is a result of downstream ER signaling and not directly related to estradiol expression. Taken together, these data suggest interaction between GFAP and estrogen results in altered astrocytic expression and has implications in the proliferation of RGCs.

### Paired box protein—pax-6

The transcription factor Pax-6 is essential in neurogenesis. Mutations in the *PAX6* gene can cause serious developmental disorders such as aniridia, autism spectrum disorder, and intellectual disability (Davis et al., 2008). Pax-6 and estradiol have been targeted in a large portion of breast cancer research. Concordantly, suppression of *PAX6* gene expression inhibits cell growth, which appeared to have a knock-on effect from or to ERK1/2, p38 and cyclin D1 (Zhao et al., 2014). In ER-positive cancer cell lines, MCF-7, Pax-6 knock-down interrupts cell cycling at G1 phase and reduced proliferation (Zong et al., 2011). Estradiol's stimulation of *PAX6* has been noted in MCF7 cell lines. Estradiol is able to stimulate the transcription activity of 187 transcription factors (Li et al., 2014b), which impresses its involvement in proliferation, migration and cell cycle regulation. Based on these data, it is plausible that Pax-6 may be the facilitator for estradiol-mediated proliferation, which can be seen in the increased proliferative status of GFAP+ NSCs (Figure 6). One might expect that Pax-6 knockdown models may be rescued by simulation of ERß during neurodevelopment. However, one must be cautious about the interpretation of this data. Purely due to the availability the authors have used evidence and data from MCF7 (Cancer) cultures, which are not directly comparable to neural circuits and cultures. However, it is the opinion of the authors that the MCF7 lines still suggest an interesting case for the interaction of estrogen and Pax-6.

### Neurogenin (Ngn)

Intracellular progenitor Notch signaling plays a pivotal role in progenitor maintenance, cell fate and differentiation (Ohtaka-Maruyama and Okado, 2015). Balancing between Hedgehog and Notch signaling maintains correct cortical formation (Dave et al., 2011). Estradiol is able to mediate the regulators of Notch signaling via ERs (Bender et al., 2010). Through the utilization of Ngn3 and GPER1, estradiol promotes neuritogenesis in the mouse hippocampus (Figure 6), which also incurred the activation of PI3K signaling (Ruiz-Palmero et al., 2013). Ngn3 is promoted through downregulation of Notch signaling (Arevalo et al., 2011). Furthermore, neurogenins could be partially responsible for sex-dependent dimorphisms. Ngn3 activity has been suggested to influence sex-dependent differentiation in neurons, which may result from epigenetic regulation of sex-linked genes (Scerbo et al., 2014). Ngn3 is also repressed by estradiol-mediated inhibition of Hes-1, which is regulated through Notch signaling (Salama-Cohen et al., 2006). Ngn2 and Ngn3 have a role in division within the VZ, which is mediated by *Hes-1*. Through which, estradiol may act as a mediator control asymmetric division. Furthermore, as Ngn3 is able to stimulate the growth and migration of cells toward the CP, it is plausible that estradiol works through Ngn3 during neurodevelopment.

### Kinases

Estradiol targets CDK Cdc25A to promote growth during cell cycling, which also influences expression of p21 and p27 (Foster et al., 2001). In cultured cortical neurons, activation of ERβ resulted in the phosphorylation of kinase proteins: p21-activated kinase and ERK1/2 Kinase proteins are responsible for regulation of actin cytoskeleton dynamics (Srivastava et al., 2010). Rapid estrogenic signaling mediated through ERα (Sanchez et al., 2009) activates Src/Rho/Cdk5, WAVE1 pathway and a RhoA/ROCK-2/Moesin cascade, which cause the protrusion of dendritic spines (Polleux and Snider, 2010; Srivastava et al., 2013b). It has been suggested that estradiol is a modulator of these processes (Srivastava et al., 2010; Sellers et al., 2015b; Zhao et al., 2017). Other CDKs have been implicated in cell cycle regulation. E-Cdk2 has been identified as a central component of estradiol's regulatory system during G1 to S phase of the cell cycle progression (Doisneau-Sixou et al., 2003). Upregulation of CDK inhibitor p21 can stimulate proliferation of embryonic stem cells induced by epidermal growth factor, which is activated by estradiol (Brännvall et al., 2002). Interaction with CDK inhibitors such as p27 can regulate ERs and *vice versa* to mediate transcription and gene expression (Prall et al., 1997). In the MCF-7 cell line ERα is mediated by p27 to regulate nuclear localisation and therefore transcription (Jeon et al., 2012). Estradiol is also able to utilize the ubiquitin proteasome system (UPS) to degrade p27, after phosphorylation by MAPK/ERK (Singer et al., 1999; Huang et al., 2012). This may serve as a regulatory mechanism for proliferation. Estradiol elicits rapid responses, which can be attributed to kinase phosphorylation (Srivastava et al., 2013b). These rapid actions can be attributed to involvement of tyrosine kinases (Karthikeyan and Thampan, 1996). Furthermore, by binding to cytoplasmic or membrane-bound receptors, estradiol is able to activate phosphorylation cascades by ERK, PI3K and MAPK (Singh et al., 1999; Fox et al., 2009).

### Small non-coding RNAs

In MCF-7 cell lines, estradiol's influence on small non-coding microRNA (miR) is well documented (Klinge, 2009; Jiang et al., 2016). Estradiol-stimulated proliferation can be induced by miR-26 in MCF-7 cell lines (Tan et al., 2014). Conversely, this process can be negatively regulated by estradiol through upregulation of miR-9, which inhibits proliferation and migration (Fang et al., 2015). Interestingly, whereas Tan's group (Tan et al., 2014) showed miR-26 was ER-dependent to induce proliferation, Fang et al.'s group (2015) showed miR-9 inhibited proliferation and migration independently of ERs (Figure 6). Current literature shows ERα induces proliferation and ERß induces apoptosis, which is reflected in miR interaction with ERα and ERß leading to polarizing effect (Helguero et al., 2005). miR-22 (Pandey and Picard, 2009), miR-221-222 (Di Leva et al., 2010), miR-206 (Adams et al., 2007; Kondo et al., 2008) are generally correlated with a reduction or inhibition of ERα and its effects. However, some miRNAs have shown to be an exception to this. miR-342 (He et al., 2013) overexpression upregulated MCF-7 cells to induce apoptosis and inhibited proliferation, which possibly highlights the interdependence of the two ERs in conditions such as extreme overexpression, which would coincide with the “ying-yang” relationship ERs have been suggested to possess (Lindberg et al., 2003).

In a clinical setting, estradiol may act on miR to account for the gender disparity seen in the diagnosis of schizophrenia (Mellios et al., 2012). Specifically, in the frontal cortex of schizophrenic male mice, miR-30b was expressed in greater quantity than in females. Many of the studies cited in this section are from cancer cell lines, which leave conclusions drawn little more than conjecture. The strength of the individual results coupled with the correlative observations, implicate miR as a player during cortical development.

Using miRs in studying cortical development will require further development of laboratory techniques. Currently, processes involved in analyzing miRs are costly and the literature has shown to be divided in agreeing upon various sequences and expression patterns (Guo et al., 2016).

### Wnt

Wnt signaling coordinates the progeny of progenitor cells during differentiation (Kriska et al., 2016), which could serve as a mechanism used by estradiol to mediate differentiation. Through Wnt3A, estradiol receptor signaling is able to promote differentiation in mesenchymal progenitor cells (Gao et al., 2013). Wnt signaling is also known to regulate neuronal differentiation of cortical intermediate progenitors (Munji et al., 2011). Cross talk between Wnt and estradiol is facilitated by interaction with ß-catenin (Kouzmenko et al., 2004). A recent review highlights the importance of estradiol in Wnt signaling as a chief mediator in maintaining the balance of Wnt antagonist Dkk1 and Wnt/ß-catenin (Scott and Brann, 2013).

### RhoA

As a key regulator of cell orientation in the cortex, RhoA activity is essential in cellular migration. Consequently, excess and deficit can cause impairment, however, ERα may serve as a regulator to RhoA activity and stability. Inactivation of ERα by siRNA increased RhoA protein expression, which resulted in decreased migration (Sailland et al., 2014). It is worth noting that PCR showed this process was independent of Wnt11 and N-Cadherin expression, which are established mechanisms leading to ERα activation (Dwyer et al., 2010). RhoA was also shown to decrease the transcriptional activity of ERα via RhoGDI increasing transactivation by Rho GTPase (Su et al., 2001).

### Cadherin

Cadherins are used to anchor the end-feet of RGCs to the ventricular surface, which is a key aspect of maintaining polarization in the developing cortex (Figure 6). In ERßKO mice, migration, morphology and polarization are defective. Furthermore, it was shown that these processes could occur as ERßKO lacked the modulation of ERß over E-Cadherin and N-Cadherin (Xu et al., 2015).

### BDNF and TrkB

The steroid hormone estrogen and the neurotrophin BDNF have been observed sharing mutual functions, in a co-dependent or even synergistic manner (Scharfman and Maclusky, 2005; Numakawa et al., 2010; Srivastava et al., 2013a). Respectively, they are established as two extracellular signaling molecules that work to meet numerous physiological functions. Estrogen and BDNF are both able to influence signaling cascades, synaptic structures and neuronal physiology in multiple and mutually inclusive areas (Greenberg et al., 2009; Waterhouse and Xu, 2009; Srivastava et al., 2010, 2011).

The connection between BDNF and estrogen is likely through a novel signaling system completely removed from the traditional trkB pathway. This is evidenced by no significant change in trkB mRNA or protein noted in gonadectomized or estrogen-replaced animals (Solum and Handa, 2002). Solum and Handa (2002) concluded that due to co-localisation of ERα and BDNF in pyramidal cells of the CA1 and CA3 hippocampal sub-regions. Since the publication of the Solum paper, other teams have uncovered a similar mechanism using GPER in the same cerebral region (Briz et al., 2015). Both groups concluded that estrogen and BDNF are in some way used to regulate cerebral function.

A possible mechanism for the interaction between BDNF and estrogen could be met through intracellular phosphorylation cascades. Estrogen has been documented providing restorative effects to ovariectomised mice. Estrogen (0.1 mg/kg) worked through PI3K/Akt signaling pathway to provide short-term neuroprotection of neurons in the midbrain by upregulating BDNF (Yi et al., 2016). Cultured dentate gyrus neurons *in vitro* reacted in comparable manner, as significant BDNF upregulation occurred after pharmacological exposure to estradiol (100 mM−1 μM) (Sato et al., 2007).

A comprehensive review of the literature in this area can be found in the references list (Srivastava et al., 2013a).

### Neuroglobin

ERα regulates neuroglobin expression through genomic transcript region regulation (Guglielmotto et al., 2016). Estradiol is capable of upregulating neuroglobin through activation of ERß (De Marinis et al., 2013; Fiocchetti et al., 2015). Estradiol may utilize neuroglobin to regulate the formation of neurites during migration. Neuroglobin is found within the SVZ and IZ (Shang et al., 2006) and increases the phosphorylative state of Akt leading to neurite outgrowth (Li et al., 2014a).

### Reelin and dab1

In hippocampal slice cultures, exogenous application of estradiol causes reelin expression to increase from Cajal-Retzius cells, which is facilitated by ERs. Furthermore, aromatase activity contributes to reelin expression. Impairment of aromatase activity causes reelin expression to reduce (Bender et al., 2010). If the aromatase gene is knocked out in male mice, specific areas in the brain have been shown to change in size (Pierman et al., 2008). Moreover, this morphological impairment can somewhat be saved through estrogen and testosterone treatments. As shown in reeler mice, estradiol upregulates reelin mRNA in the Purkinje cells (PC) of the cerebellum, with a particularly notable effect in males. Reelin protein bands (420, 310, and 180 kDa) were also shown to be upregulated. Furthermore, *reeler* mice show an abnormal steroid hormone profile with testosterone and estradiol increased but dihydrotesterone decreased (Biamonte et al., 2009).

### Apolipoprotein

Estradiol down-regulates ApoER2 in differentiating osteoblasts, which is reflected in other LDLR family members. Leading to the promotion of differentiation in osteoclasts (Gui et al., 2016). Estradiol may alter apolipoprotein expression through genomic transcript mediation. Estradiol is able to upregulate ApoE gene expression in BL6 mice (Srivastava et al., 1997). ERα mRNA expression during development correlates with regulation of differentiation of osteoblasts (Bodine et al., 1998). Differentiation of apical papilla stem cells is also regulated by estradiol via the activation of MAPK signaling (Li et al., 2014c). Furthermore, ApoER2 interacts with reelin to govern morphology and cytoarchitecture (Stranahan et al., 2013).

### Estradiol and gene expression

The cortical transcriptome can be manipulated epigenetically by estradiol, including functions ranging from memory (Frick et al., 2011), transcription factor regulation (Jadhav et al., 2015), and cell cycling (Couse et al., 1995). Estradiol has shown to significantly alter at least 88 genes in relation to the control of the cortex (Humphreys et al., 2014). Genes associated with synaptic activity, myelination, synthesis and metabolism, neurotransmission and kinase signaling were all shown to alter expression following introduction of estradiol after ovariectomy. Removing the expression and synthesis of extra-nervous estrogen in males, would likely further exacerbate the effects and may highlight sex-dependent gene expression in cortical development. Introduction of estradiol in ovariectomised rats induces genomic changes that influence the dopaminergic and peptidergic neural networks (Sárvári et al., 2010). This may be as a result of estradiol's role in neurotransmission. It is known that aromatase is localized to the synapse and can be activated by calcium phosphorylation (Balthazart and Ball, 2017).

ERα has also shown to mediate subtle epigenetic mechanism that result in sex-specific differences in the prefrontal cortex in adulthood (Westberry and Wilson, 2012). It is plausible that this sex-specific difference is met through alteration in the inhibitory GABAergic signaling pathway. Pyramidal neurons of the prefrontal cortex rely on phosphorylation-dependent mechanisms for cellular trafficking and signaling. G protein-gated inwardly rectifying potassium channels modulate excitability and were shown to be susceptible to sex-specific neurochemistry (Marron Fernandez de Velasco et al., 2015). The extent to which estradiol is able to regulate these mechanisms remains to be seen. Estradiol affects the genetic transcriptome of 88 genes (likely more) within the cerebral cortex. However, questions remain: which mechanisms does estradiol utilize to interact with these genes and are these genes affected in different sexes? Are males more susceptible to estrogen transcriptome deficits during cortical development? Does the knockout of aromatase impact estrogenic signaling? Does aromatase knockout affect males and females unequally? Does the aromatase knockout affect the transcriptome? Answering these questions would prove to further elucidate the pathways involved in cortical development. Furthermore, answering these questions would further elucidate the mechanisms estrogen adopts to control sexual dimorphism and to what extent it is able to control sexual dimorphism. Delivering a clear mechanism for sexual dimorphism would also aid in better understanding of neurodevelopmental disorders that show biased sexual distribution, such as autism spectrum disorder.

## Summary and conclusions

In summary, many lines of evidence exist that suggest estradiol has many critical roles in corticogenesis (Figure 6). Hitherto, no clear mechanism has been established. Substantial evidence purports to show a connection between estradiol, migration, and neurogenesis. A strong body of evidence exists showing that estradiol influences the proliferative body within the subventricular zone, which increases the availability of NSCs. The mechanism underlying this action has not been established but may be associated with Pax-6, neurogenins, and nestin. Estradiol influences many regulators of estrogenic-signaling, which suggests that this maybe a primary function. Mechanisms underlying estradiol's influence in regulating migration has been discussed in detail. The interaction with Wnt, Cadherin as well as various kinases produce dynamic actin changes, which has been suggested as mechanism for migration. Estradiol has also shown to associate greatly with the reelin/Dab1 system. Collectively, this investigation suggests estradiol as a proliferative regulator and migratory stimulator. Disruption to estrogenic signaling may result in an enlarged SVZ from impaired migration and un-regulated proliferative cycle. Alternatively, cells may accumulate within the CP after being ejected too early from the cell cycle. Understanding these mechanisms will further benefit developmental disorders.

## Author contributions

MCSD and DPS wrote the manuscript. NJFG and KJS edited and contributed to the writing of the manuscript. KJS and DPS oversaw the project.

### Conflict of interest statement

The authors declare that the research was conducted in the absence of any commercial or financial relationships that could be construed as a potential conflict of interest.
